# Efficacy of Nivolumab in Chemotherapy‐Resistant Mesothelioma of the Tunica Vaginalis Testis: A Case Report

**DOI:** 10.1002/cnr2.70647

**Published:** 2026-08-14

**Authors:** Haruki Koyanagi, Takashi Kawahara, Natsuki Inagawa, Miho Shiga, Hiromichi Sakurai, Akane Yamaguchi, Kouzaburou Tanuma, Satoshi Nitta, Kosuke Kojo, Masanobu Shiga, Yoshiyuki Nagumo, Atsushi Ikeda, Shuya Kandori, Akio Hoshi, Hiromitsu Negoro, Hitomi Kawai, Daisuke Matsubara, Hiroyuki Nishiyama

**Affiliations:** ^1^ Department of Urology, Faculty of Medicine University of Tsukuba Tsukuba Ibaraki Japan; ^2^ Department of Pathology, Faculty of Medicine University of Tsukuba Tsukuba Ibaraki Japan

**Keywords:** mesothelioma, nivolumab, tunica vaginalis

## Abstract

**Background:**

Mesothelioma of the tunica vaginalis testis is a rare neoplasm, accounting for less than 1% of all mesothelioma cases. Due to the rarity of this disease, a standard treatment has not been established. Clinical management is generally adapted from the strategies used for pleural mesothelioma. Although immune checkpoint inhibitors (ICIs) have demonstrated efficacy in treating pleural mesothelioma, their role in mesothelioma of the tunica vaginalis testis remains uncharacterized due to a lack of evidence. This is the first report documenting a clinical response to nivolumab in chemoresistant mesothelioma of the tunica vaginalis testis.

**Case:**

A 71‐year‐old man presented with painless left scrotal swelling and was initially diagnosed with a hydrocele at the referring hospital. One year later, hydrocelectomy revealed partial thickening of the tunica without malignancy. A subsequent magnetic resonance imaging analysis revealed a new intratesticular nodule. Orchiectomy was performed, and the pathological findings revealed an epithelioid mesothelioma. He was referred to Tsukuba University Hospital for further treatment. He also had a history of Self‐Defense Force service, which suggested possible asbestos exposure. Imaging revealed pelvic lymphadenopathy with fluorodeoxyglucose uptake in the right external iliac lymph nodes. He received four cycles of cisplatin and pemetrexed every 3 weeks, resulting in stable disease. Bilateral pelvic lymph node dissection was performed and confirmed the presence of metastatic mesothelioma. Nine months after the surgery, bilateral inguinal lymph node recurrence occurred, and he received cisplatin monotherapy because of renal impairment. Despite four additional cycles of cisplatin monotherapy, the disease progressed. Therefore, chemotherapy was discontinued, and nivolumab was administered. Nivolumab resulted in sustained tumor shrinkage. However, after 3 months of treatment, he developed immune‐related encephalitis requiring corticosteroid therapy and nivolumab discontinuation. After 16 months of nivolumab initiation, he was dead from the disease.

**Conclusion:**

This is the first report documenting the clinical response of a chemoresistant mesothelioma of the tunica vaginalis testis to nivolumab. ICIs are a promising treatment option for this rare and challenging disease.

## 1 Introduction

Mesothelioma of the tunica vaginalis testis is a rare neoplasm that accounts for less than 1% of all mesothelioma cases [1, 2]. Recent studies have emphasized the rarity of this disease and the limited evidence available for its management [2]. Due to the rarity of this disease, no standard treatment has been established. Surgical resection, most commonly involving orchiectomy, is the preferred approach. In cases of metastatic or postoperative recurrence, platinum‐based chemotherapy (e.g., cisplatin plus pemetrexed) is administered, following the established protocols for pleural mesothelioma [3].

In cases of unresectable or metastatic pleural mesothelioma, the first‐line therapy typically consists of platinum‐based chemotherapy (e.g., cisplatin plus pemetrexed) or immune checkpoint inhibitors (ICIs; e.g., nivolumab plus ipilimumab), depending on the histologic subtype and patient characteristics. For patients with disease progression, second‐line options include gemcitabine, vinorelbine, and additional ICI therapy [4].

Previous reports have confirmed the clinical efficacy of ICIs in patients with pleural mesothelioma that has become resistant to conventional chemotherapy [5]. However, the efficacy of ICIs specifically in patients with mesothelioma of the tunica vaginalis remains unclear. While the efficacy of combined ICIs therapy, specifically nivolumab plus ipilimumab, has been reported in treatment‐naïve metastatic mesothelioma of tunica vaginalis testis, no previous reports have demonstrated the clinical benefits of ICIs in patients who have developed resistance to first‐line chemotherapy [6]. This is the first report documenting a clinical response to nivolumab in chemoresistant mesothelioma of the tunica vaginalis testis.

## 2 Case

A 71‐year‐old man presented with painless left scrotal swelling and was initially diagnosed with a hydrocele at the referral hospital (October/Year 2017). He was managed conservatively for 1 year. Hydrocelectomy was performed because the swelling increased (September/Year 2018). Histopathological analysis revealed partial thickening of the tunica wall. However, no definitive evidence of malignancy was obtained. Therefore, close monitoring was recommended. After 1 year of the hydrocelectomy, a magnetic resonance imaging (MRI) analysis revealed a newly developed intratesticular nodule. He was diagnosed with local recurrence of mesothelioma and underwent orchiectomy with resection of the scrotal skin and surrounding tissue (December/Year 2019, Figure 1).

**FIGURE 1:**
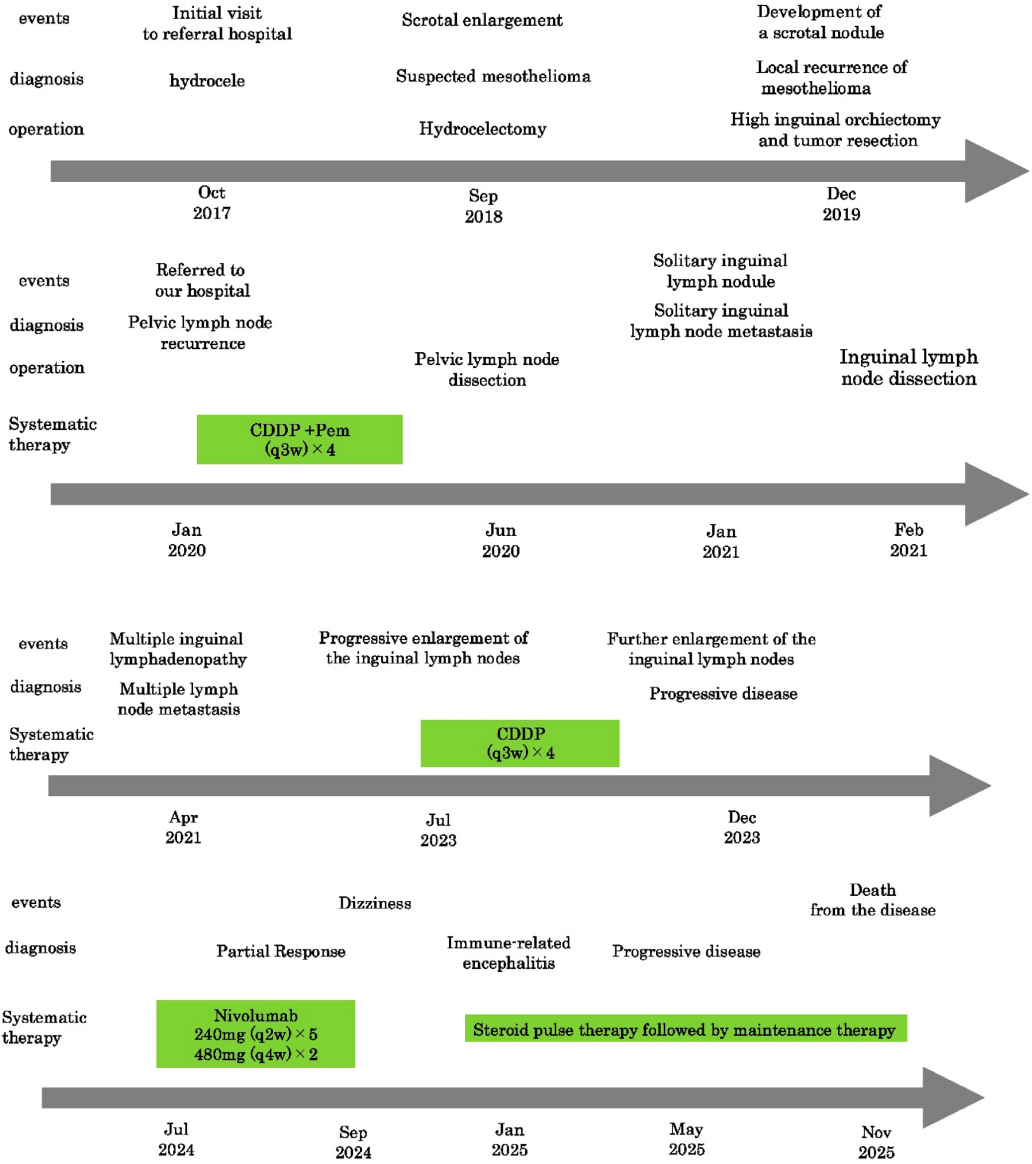
Treatment timeline of the patient with recurrent mesothelioma of the tunica vaginalis testis.

**FIGURE 2:**
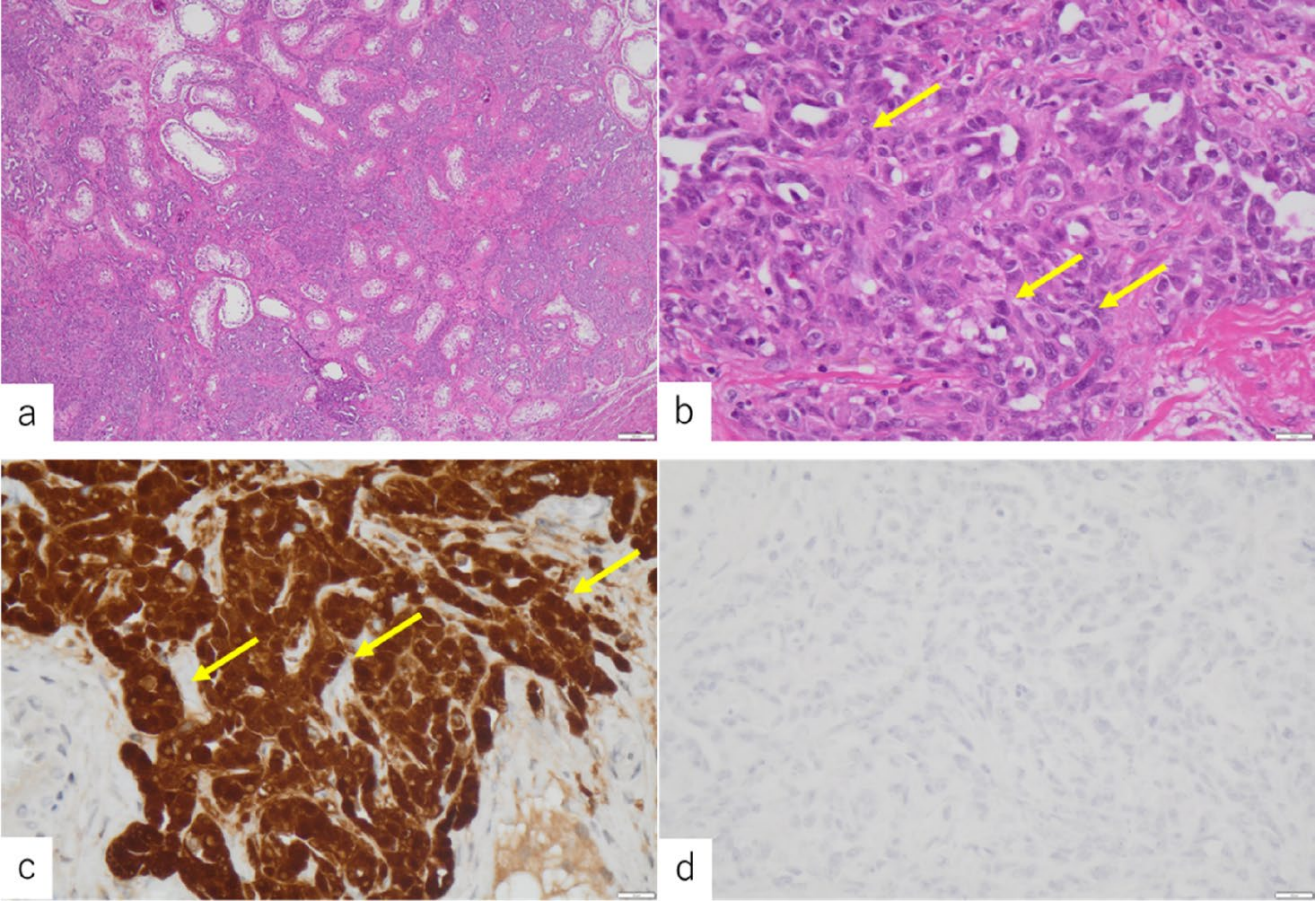
Histopathological and immunohistochemical findings of the resected testicular tumor. (a) Hematoxylin and eosin (H&E)‐stained sample; ×4. (b) H&E‐stained sample; ×40. (c) Calretinin immunohistochemical staining at ×40. (d) Carcinoembryonic antigen (CEA) immunohistochemical staining at ×40.

Histologically, atypical cells with eosinophilic cytoplasm proliferated in papillary and nested patterns, involving the testis and surrounding connective tissue (Figure 2a,b). Immunohistochemically, the tumor was positive for calretinin and negative for carcinoembryonic antigen (CEA) (Figure 2c,d). These findings were consistent with epithelioid mesothelioma. He was referred to Tsukuba University Hospital for further management. He had a history of service in the Self‐Defense Force, which suggested the possibility of asbestos exposure.

Computed tomography (CT) revealed pelvic lymphadenopathy, and positron emission tomography‐CT (PET‐CT) showed fluorodeoxyglucose (FDG) uptake in the right external iliac lymph nodes (Figure 4a). He was diagnosed with a pelvic lymph node recurrence (January/Year 2020). Combination chemotherapy with cisplatin (75 mg/m^2^ on Day 1) and pemetrexed (500 mg/m^2^ on Day 1) was administered every 3 weeks. Four cycles of combination chemotherapy were completed. Subsequently, CT showed stable disease. Bilateral pelvic lymph node dissection was performed, which revealed metastatic mesothelioma (June/Year 2020).

**FIGURE 3:**
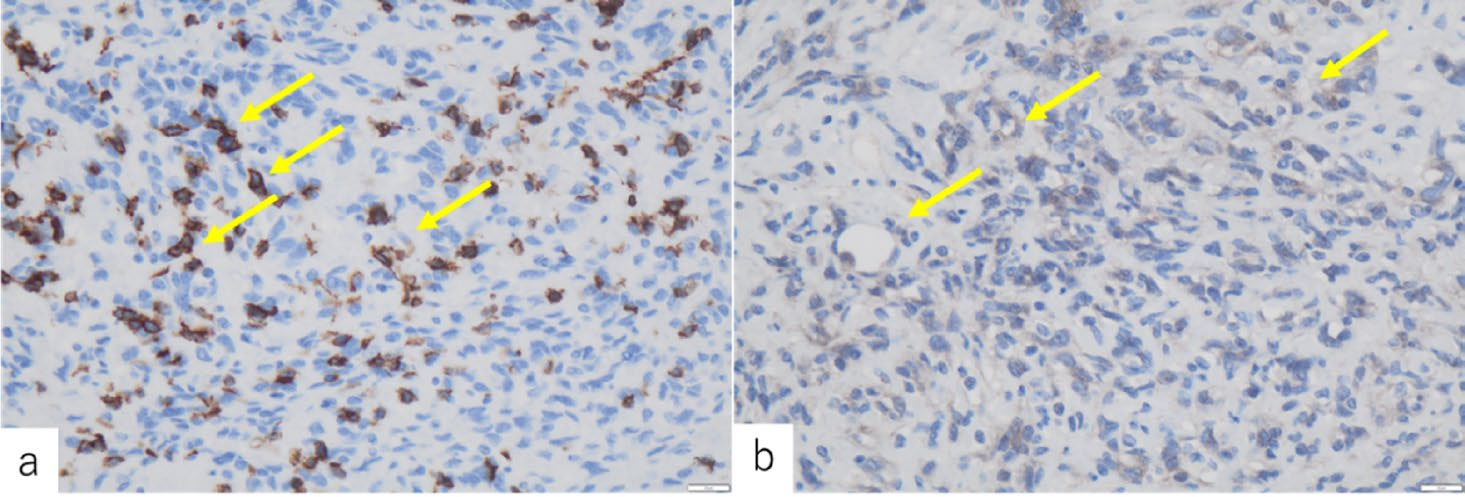
Histopathological and immunohistochemical findings of the resected lymph node. (a) CD8^−^ immunohistochemical staining at ×40. (b) PD‐L1 immunohistochemical staining at ×40.

Six months later, a solitary left inguinal lymph node enlargement was noted (January/Year 2021). Dissection was performed, and mesothelioma metastasis was confirmed (February/Year 2021). Additional immunostaining demonstrated the mild infiltration of CD8^+^ T cells (Figure 3a) and PD‐L1 positivity (Figure 3b). Three months after the left inguinal lymph node dissection, bilateral inguinal lymph node recurrence occurred (April/Year 2021, Figure 4b).

**FIGURE 4:**
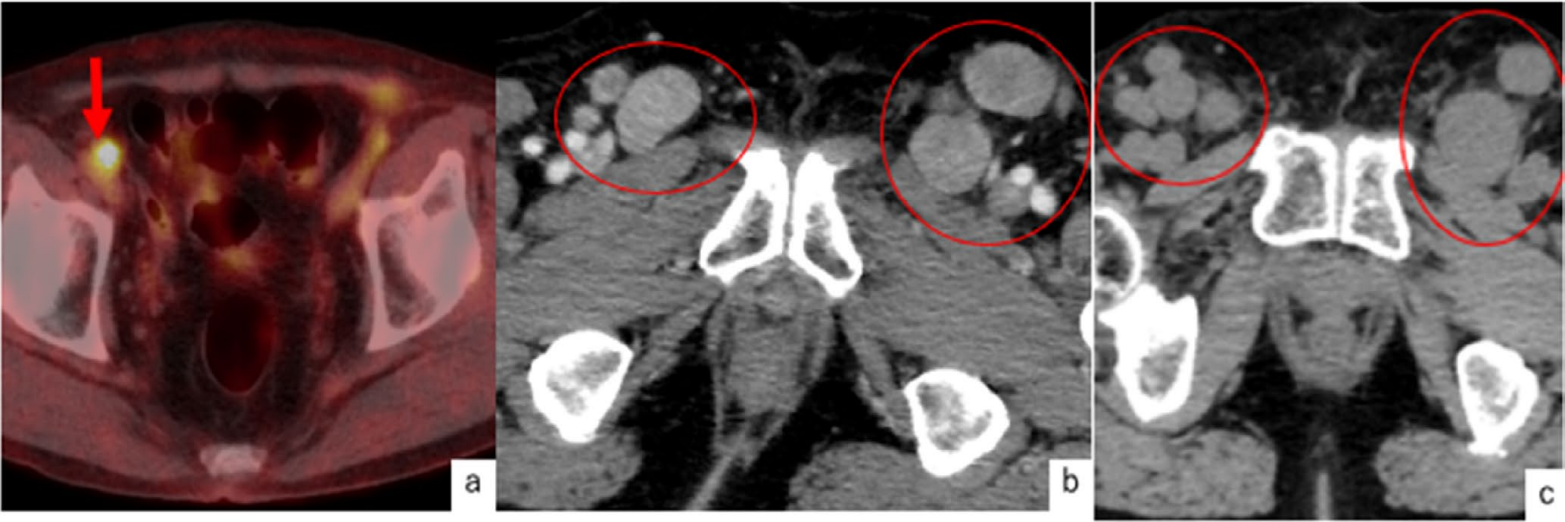
FDG‐PET at metastatic diagnosis (a), CT before chemotherapy (b), and after chemotherapy (c).

Mild symptoms were observed; however, as the lesions enlarged and the symptoms worsened, cisplatin monotherapy was restarted because of renal impairment (July/Year 2023). Despite completing four cycles of cisplatin monotherapy, the disease progressed, and treatment was discontinued (December/Year 2023, Figure 4c).

**FIGURE 5:**
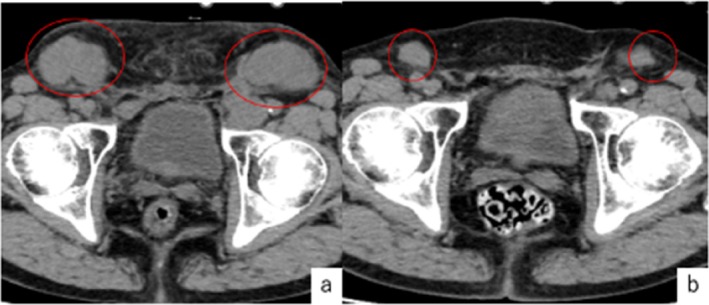
Radiological response of lymph node metastases to nivolumab on CT: Before treatment (a) and after treatment (b).

After nivolumab therapy for nonpleural mesothelioma was covered by insurance in Japan, nivolumab treatment was initiated (July/Year 2024). Nivolumab efficacy was determined according to RECIST ver. 1.1, and safety profiles were graded according to the CTCAE ver. 5. Nivolumab was administered at a dose of 240 mg every 2 weeks for five cycles, followed by 480 mg every 4 weeks for two additional cycles. Two months after nivolumab initiation, the bilateral inguinal lymph node metastasis decreased in size from 3 to 2 cm without the appearance of any new lesions, indicating a partial response (PR) (Figure 5a,b). Three months after initiation of nivolumab therapy, he developed dizziness (September/Year 2024). Based on MRI findings, he was diagnosed with immune‐related encephalitis (irAE) categorized as Grade 3.

Nivolumab was discontinued, and high‐dose corticosteroid therapy was initiated, consisting of methylprednisolone at 1000 mg/day for 3 days as pulse therapy, followed by oral prednisolone at 25 mg/day, resulting in a reduction in symptoms (January/Year 2025). The inguinal lymph nodes enlarged subsequently with no other lesions identified (May/Year 2025). Rechallenge with nivolumab was considered; however, after consultation with neurologists, it was withheld due to the perceived high risk of recurrent encephalitis. Inguinal lymph node metastases progressed, with the development of subcutaneous metastases and ascites (October/Year 2025). After 16 months of nivolumab initiation, he was dead from the disease (November/Year 2025).

## 3 Discussion

To the best of our knowledge, this is the first report to demonstrate a clinical response to an ICI in chemotherapy‐resistant mesothelioma of the tunica vaginalis testis. Mishra et al. [6] reported the use of nivolumab plus ipilimumab as first‐line treatment in a patient with metastatic mesothelioma of the tunica vaginalis testis, resulting in a PR that was sustained for 6 months. However, this case involved chemotherapy administration before ICI. Moreover, no previous report has documented the response to immunotherapy in a patient with disease progression following platinum‐based chemotherapy. Considering the rarity of this disease and the absence of standard second‐line therapies, our case provides novel evidence that single‐agent nivolumab may be a promising therapeutic option in cases of heavily pretreated mesothelioma of the tunica vaginalis testis. However, the existing literature has shown no definitive benefit.

Asbestos exposure is widely recognized as a significant risk factor for mesothelioma, and 69%–93% of pleural mesothelioma cases in Europe and America have reportedly been associated with asbestos exposure [5, 7]. However, a history of asbestos exposure has been documented in 34%–66% of patients with mesothelioma of the tunica vaginalis testis [1, 2, 3, 8]. In this case, the patient's occupational history as a former Self‐Defense Force member suggested possible asbestos exposure. The prognosis of mesothelioma of the tunica vaginalis remains poor. The median overall survival is approximately 24 months, with a shorter survival time of approximately 14 months in cases of recurrence [1, 2, 3]. The disease typically presents as a painless scrotal swelling or hydrocele, often mimicking benign conditions and contributing to diagnostic delays [1]. In the present case, the initial pathological diagnosis was inconclusive, and a definitive diagnosis was made following disease progression. Immunohistochemical analysis revealed positive results for calretinin and CK5/6, which are consistent with typical mesothelioma profiles [4].

Due to the rarity of this disease, no standardized treatment protocols are available. Management is typically extrapolated from the treatment guidelines for pleural mesothelioma, for which platinum‐based chemotherapy remains the first‐line treatment [9]. In the present case, after determining that the disease was unresectable, platinum‐based combination chemotherapy was initially considered in accordance with the treatment strategies for pleural mesothelioma. However, owing to renal impairment, cisplatin monotherapy was selected as a safer alternative.

However, most patients eventually progress, and no second‐line treatment has been established. As noted by Drevinskaite et al. [10], the systemic therapy for chemoresistant mesothelioma of the tunica vaginalis testis remains largely unknown. Although second‐line treatments, such as gemcitabine or vinorelbine, have been reported (Table 1), few cases with poor outcomes have been documented.

**TABLE 1:** Summary of reported cases of mesothelioma of the tunica vaginalis testis [10, 11, 12].

Reporter (date)	Age	Sites of metastatics	First line	Second line	Overall survival after second line
Okutani (1988) [11]	53	Right femoral arterial lymph node Primary tumor in the perineal region Thoracic vertebra	Cyclophosphamide + 5‐FU + vincristine + cisplatin	Cyclophosphamide + cisplatin + adriamycin	Dead (1 month)
Drevinskaite (2020) [10]	69	Paraaortic lymph nodes	Cisplatin + pemetrexed	Gemcitabine	Dead (3 months)
Rathinasamy (2022) [12]	68	Hilar lymph nodes	Cisplatin + pemetrexed	Gemcitabine	Dead (10 months)
Rathinasamy (2022) [12]	68	Lung	Cisplatin + pemetrexed	Gemcitabine	Alive (21 months)
Rathinasamy (2022) [12]	50	Intrathoracic lymph nodes	Cisplatin + pemetrexed	Vinorelbine	Dead (13 months)
Present case	78	Inguinal lymph nodes	Cisplatin + pemetrexed	Nivolumab	Dead (16 months)

Recently, ICIs have shown a survival benefit in patients with pleural mesothelioma. The MERIT trial demonstrated the efficacy and safety of nivolumab in patients with pleural mesothelioma that progressed after chemotherapy and the CONFIRM trial further established that nivolumab improves overall survival of previously treated patients [5, 13]. In the management of pleural mesothelioma, immunotherapy constitutes a primary first‐line strategy; furthermore, it demonstrates a good clinical benefit in chemoresistant cases [14]. However, its efficacy in treating mesothelioma of the tunica vaginalis testis has not yet been demonstrated. Our case raises the possibility that ICIs have therapeutic potential, even in this rare setting.

Despite a favorable response, the treatment was discontinued because of irAE, a rare but serious adverse event. This finding underscores the importance of careful monitoring, especially in older patients. As ICIs are increasingly being used for the treatment of rare tumors, clinicians must compare their benefits with the potential immune‐related toxicities. Evidence accumulation from additional cases and prospective studies is needed to define the efficacy and safety in this context.

In summary, we reported a rare case of metastatic mesothelioma of the tunica vaginalis testis that progressed despite administration of standard platinum‐based chemotherapy. The disease responded to nivolumab. To our knowledge, this is the first report demonstrating a clinical response to immune checkpoint inhibition in chemotherapy‐resistant mesothelioma of the tunica vaginalis testis. This rare entity lacks standardized treatment strategies beyond platinum‐based chemotherapy, and no prior literature has confirmed the clinical efficacy of ICIs in this setting. Reports demonstrating the efficacy of nivolumab in treatment‐naïve mesothelioma of the tunica vaginalis testis are limited; therefore, conventional chemotherapy should currently remain the priority for first‐line treatment. Our case highlights the potential role of nivolumab in achieving tumor regression, thereby suggesting that ICIs may serve as a viable salvage option in select patients with pretreated disease. This case highlights the urgent need for collaborative, multicenter efforts and prospective data collection to better characterize the clinical course, treatment responsiveness, and toxicity profiles of ICIs in mesothelioma of the tunica vaginalis testis.

## 4 Conclusion

This is the first report documenting the clinical response of a chemoresistant mesothelioma of the tunica vaginalis testis to nivolumab. While ICIs are a promising treatment option for this rare and challenging disease, clinicians must remain vigilant regarding potentially severe immune‐related toxicities. Further studies and case reports are needed to establish the definitive efficacy and safety of ICIs in this clinical setting.

## Author Contributions

**Masanobu Shiga:** supervision. **Takashi Kawahara:** conceptualization, writing – original draft, writing – review and editing. **Akane Yamaguchi:** data curation. **Yoshiyuki Nagumo:** supervision. **Natsuki Inagawa:** data curation. **Satoshi Nitta:** supervision. **Miho Shiga:** data curation. **Kosuke Kojo:** supervision. **Kouzaburou Tanuma:** data curation. **Haruki Koyanagi:** writing – review and editing, writing – original draft, conceptualization. **Atsushi Ikeda:** supervision. **Shuya Kandori:** writing – review and editing. **Daisuke Matsubara:** resources. **Hiromitsu Negoro:** writing – review and editing. **Hiroyuki Nishiyama:** writing – review and editing. **Akio Hoshi:** writing – review and editing. **Hitomi Kawai:** resources. **Hiromichi Sakurai:** data curation.

## Funding

The authors have nothing to report.

## Ethics Statement

Ethics approval was waived for this case report, as it describes a single patient's clinical course without experimental intervention.

## Consent

Written informed consent for publication had already been obtained from the patient’s son (the next of the kin) in the presence of the patient while the patient was still alive, prior to his death in 2025.

## Conflicts of Interest

The authors declare no conflicts of interest.

## Data Availability

Data sharing not applicable to this article as no datasets were generated or analysed during the current study.
